# TROP2 modulates the progression in papillary thyroid carcinoma

**DOI:** 10.7150/jca.62461

**Published:** 2021-09-27

**Authors:** Huali Sun, Qi Chen, Weiping Liu, Yanmei Liu, Sihan Ruan, Chumeng Zhu, Yanyun Ruan, Shenpeng Ying, Peipei Lin

**Affiliations:** 1 Department of Radiotherapy, Taizhou Central Hospital (Taizhou University Hospital), Taizhou, Zhejiang, P. R. China.; 2 Precision Medicine Center, Taizhou Central Hospital (Taizhou University Hospital), Taizhou, Zhejiang, P. R. China.; 3 Nuclear Medicine Department, Taizhou Central Hospital (Taizhou University Hospital), Taizhou, Zhejiang, P. R. China.

**Keywords:** TROP2, ISG15, Progression, Papillary thyroid carcinoma, Immunization

## Abstract

**Background:** Tumor-associated calcium signal transducer 2 (TROP2) is over expressed in various kinds of human cancers and plays important roles in the proliferation, invasion and metastasis of tumor cells. However, the expression and molecular mechanism of TROP2 in thyroid papillary carcinoma (PTC) are unclear.

**Methods:** The expressions of TROP2 in PTC and control tissue were detected by real-time reverse transcription polymerase chain reaction (RT-PCR) and immunohistochemistry. The proliferation and invasion of PTC cell lines were examined by cell cloning and transwell assays. RNA sequencing analysis and public data analysis were assessed to investigate the potential mechanisms of TROP2 in PTC. Gene correlation analysis was conducted to explore the association between TROP2 and the related gene ISG15 in patients with PTC.

**Results:** The expression of TROP2 was significantly higher in PTC than control. The high expression of TROP2 protein was associated with lymph node metastasis, tumor size and capsular infiltration (P<0.05). SiRNA-mediated TROP2 gene expression silencing can significantly inhibit proliferation and migration of PTC cells. ISG15 decreased in TROP2 siRNA PTC cells and increased in PTC patients significantly. There was a significant correlation between the expression of TROP2 and ISG15 in PTC patients. TROP2 interacted directly with ATP6V1A, CEBPA and SOX5 and then further interacted with the immune genes. TROP2 expression and tumor-infiltrating immune cells were also correlated in thyroid cancer microenvironment.

**Conclusions:** TROP2 promotes the development of PTC. TROP2 expression was correlated with ISG15 and tumor-infiltrating immune cells in thyroid cancer.

## Introduction

Thyroid cancer is the most common malignant tumor in endocrine system. In recent years, the incidence rate of thyroid cancer has increased dramatically with an average annual increase of 5.4% to 6.5%. Papillary thyroid carcinoma (PTC) accounts for 80%-90% of all differentiated thyroid carcinomas. Between 2000 and 2017, the incidence rate of adult PTC increased from 7.9/100000 to 16.9/100000 1. However, the pathogenesis of PTC is unclear 2.

TROP2 (Trophoblast cell-surface antigen 2, also known as EGP-1 or GA733-1) is called tumor-associated calcium signal transducer 2 (TACSTD2). It is a transmembrane glycoprotein encoded and expressed by TACSTD2 gene on chromosome 1p32, with a molecular weight of about 36KD 3-5. The primary structure of TROP2 protein consists of 323 amino acids, including hydrophobic leading peptide (AA1-26), extracellular domain (AA27-274), transmembrane domain (AA275-297) and cytoplasmic tail (AA298-323). Moreover, the TROP2 protein N-terminal is an extracellular domain (TROP2 EC), which is connected to the intracellular residue (trop2 IC) through a unidirectional transmembrane helix (TM), and TROP2 is fixed with the cell membrane. TROP2 has been confirmed to be highly expressed in various malignant tumors, such as ovarian carcinoma, cervical cancer 6-10, lung cancer 11,12, pancreatic cancer 13 and prostate cancer 14. In addition, TROP2 is related to the occurrence, development, invasion and clinical prognosis of tumors 15-17. However, there are few reports on the role and regulatory mechanism of TROP2 in the occurrence and development of PTC.

In the current study, the role of TROP2 in the development and progression of thyroid cancer was investigated. At the same time, the production of TROP2 in thyroid cancer was evaluated and its possible mechanism was discussed.

## Methods

### Patients and Materials

From January 2018 to December 2018, 60 PTC specimens and matched adjacent tissues were collected from Taizhou central hospital. Those PTC patients (aged 18-70 years) were confirmed by pathology. They had no tumor related diseases or received anti-tumor treatment before operation. The normal tissue was obtained 3 cm from the edge of PTC without any tumor cells confirmed by specialized pathologists. All samples were frozen in liquid nitrogen after collection. All tissue samples were informed and agreed by the patients and ethically approved by the Institutional Research Ethics Committee (application No. 2017-101).

### Real-time reverse transcription polymerase chain reaction

About 50 mg specimens of thyroid papillary carcinoma and adjacent tissues were collected. Total RNA was isolated from tissues according to the instructions of the kits TRIzol™ (Abcam, UK) or GenElute™ Total RNA Purification Kit (Abcam, UK). The concentration of RNA was determined by measuring absorbance at 260 nm. The radio of the readings at 260 nm and 280 nm provided an estimate of RNA purity. The A260/A280 ratio of RNA was 1.9-2.0. The RNA degradation and contamination was monitored by 1% agarose gels. After the agarose was prepared, the electrophoresis was carried out. The RNA products were transformed into cDNA reaction system and incubated at 37 °C for 60 minutes. Then 40 real-time PCR cycles were carried out with SYBR green fluorescent dye according to the instructions of the kit (Wenzhou Changfeng Biotechnology Co. Ltd. ZheJiang. China). Finally, the fluorescence signal was collected and its expression levels was calculated by 2^-ΔΔCt^ method. The primers selected were as follows: TROP2 forward, 5'-ACAACGATGGCCTCTACGAC-3'; TROP2reverse, 5'-GTCCAGGTCTGAGTGGT TGAA-3'.

### Immunohistochemistry

Thyroid papillary carcinoma and adjacent non neoplastic thyroid tissues, which were soaked in formaldehyde with a thickness of 4µm, were detected by immunohistochemistry (IHC). The slide was roasted at 68 °C for 2 hours, and then dipped in xylene for 20 minutes with 3 times. Samples were incubated using primary antibody (TROP2 antibody concentration 1:100; Abcam, UK) at 4 °C for 12 hours. The immunostain was tested after adding the secondary antibody (dilution 1:50; Dako, Denmark) and placed at 37 °C for for one hour. Then samples were treated with the horseradish peroxidase (HRP)/3,3'-diaminobenzidine (DAB) kit (ZSGB-BIO, China) for 2 minutes. The result of TROP2 protein was interpreted according to the conventional pathological method. Two experienced pathologists used semi-quantitative method to analyze and judge the results, and took the average value of their results obtained. The staining results were semi-quantified by any of the following scores: No staining, 0; Pale yellow, 1; Tan, 2; Brown, 3; Nuclear staining in 0-25% of cells, 0; Nuclear staining in 25-50% of cells, 1; Nuclear staining in 50-75% of cells, 3; Nuclear staining in 75-100% of cells, 4. The final IHC score was obtained by multiplying the staining color score by the nuclear staining proportion score.

### Western-blotting

100 mg of fresh frozen 60 cases of thyroid papillary carcinoma and adjacent tissues were sliced and sonicated with protein lysis buffer. The protein extracts (30-40 μg) were separated by 10% SDS polyacrylamide gel. The total proteins were separated by 10% SDS polyacrylamide gel, then separated by SDS-PAGE and transferred to PVDF membranes (Millipore, USA). After blocking with 5% non-fat milk in TBS-T for 1.5 hours, the membranes were treated with the primary antibodies of TROP2 (concentration 1:1500; Abcam, UK) or β-actin (concentration 1:3000; Abcam, UK) at 4 °C overnight. The membranes were incubated with the secondary antibody at an appropriate concentration for 1.5 hours. The protein band was observed by enhanced chemiluminescence detection system (Abcam, UK) and the gray value of protein was calculated.

### Small interference RNA (siRNA)

The cells were kept in an incubator under an atmosphere of 5% CO_2_ at 37 °C. Cell transfection was performed using Lipofectamine2000 (Thermo Fisher Scientific, US), with 40 pmol/mL TROP2-siRNA, or negative control-siRNA or the fluorescent dye CY3-NC sequence (Genepharma, Shanghai, China) in accordance with the manufacturer's protocol. The RNA was extracted after 48 hours and the protein was extracted after 72 hours to detect the interference effect. The TROP2 siRNA interference sequences were as follows: TROP2 siRNA-1, 5'-CCAAGUGUCUGCUCAATT-3', 5'-UUGAGCAG CGACACUUGGTT-3'; TROP2 siRNA-2, 5'-GGCAGAACACGUCUCAGAATT-3', 5'-UUCUGAGACGUG UUCUGCCTT-3'. TROP2 siRNA-2 was used in further experiments, such as transwell assays and wound healing assays.

### Cellular viability assays

Exponentially growing thyroid cancer cells (KTC-1 and CGTHW-3 cells) were seeded in 96-well plates with a density of 2 × 10^3^ cells per well (100 μL). After 24 hours, the siRNA transfection mixture was added to the cell culture plates. Then, after 48 hours, MTT (Promega, USA) reagent was added to each well and incubated in an incubator at 37 °C for 2 hours under 5% CO_2_. Finally, 150 uL dimethylsulfoxide (DMSO) were added to each well. The OD value of each well was measured at the wavelength of 450 nm.

### Clone plate experiment

Exponentially growing CGTHW-3 cells and TROP2 siRNA CGTHW-3 cells were collected. Cells suspension was prepared after 0.25% trypsin digestion. Each cell culture was inoculated with 100 CGTHW-3 cells and TROP2 siRNA CGTHW-3 cells. And, those cells were cultured at 37 °C in an incubator for 2-3 weeks under 5% CO_2_. The culture was stopped after cloning the cells. Cells were fixed with 4% formaldehyde for 15 minutes and stained with GIMAS.

### Transwell assays

The transfection thyroid cancer cell lines (KTC-1 and CGTHW-3 cells) were plated at a concentration of 2.5×10^5^/mL. Then, 200 µL serum-free medium was added to the upper chamber. And, 600 µL medium containing 10% fetal bovine serum (FBS) was added to the lower chamber. Subsequently, KTC-1 and CGTHW-3 cells (5×10^4^/well) was added to the upper chamber and incubated. After 24 hours, the chamber was removed and cleaned with PBS. The samples were further fixed with 4% fresh formaldehyde and stained with 0.1% crystal violet. After 20 minutes, the upper unmigrated cells were then wiped, cleaned, air-dried, photographed and counted. The cells passing through the filter were imaged at 100 × magnification in six random fields, and measured using the Image J software (National Institutes of Health and the Laboratory, Bethesda, MD, USA).

### Wound healing assays

About 5×10^5^ thyroid cancer cells (KTC-1 and CGTHW-3 cells) and TROP2 siRNA thyroid cancer cells were added to the 6-well plates. After the cells grew to 90%, the bottom of the 6-well plate was scratched with a 10 µl tip. After incubation at 37 °C under 5% CO_2_ for 24 hours, the 6-well plates were photographed.

### Gene expression profiling analysis

The TROP2 and negative siRNA KTC-1 cells were used for RNA sequence (Novogene Company, Tianjin, China). The total RNA amount of each sample used as input material is 1 µg for the RNA sample preparation. Sequencing libraries were generated using NEBNext® UltraTM RNA Library Prep Kit for Illumina® (NEB, USA) following manufacturer's recommendations and index codes were added to attribute sequences to each sample. Briefly, mRNA was purified from total RNA using poly-T oligo-attached magnetic beads. Fragmentation was carried out using divalent cations under elevated temperature in NEBNext First Strand Synthesis Reaction Buffer (5X). The first strand cDNA was synthesized using random hexamer primer and M-MuLV Reverse Transcriptase (RNase H-). The second strand cDNA synthesis was subsequently performed using DNA Polymerase I and RNase H. The remaining overhangs were transformed into blunt ends by exonuclease/polymerase activities. After adenylation of 3' ends of DNA fragments, NEBNext Adaptor with hairpin loop structure were ligated to prepare for hybridization. In order to screen cDNA fragments with length between 250 and 300 BP, the library fragments were purified with AMPure XP system (Beckman Coulter, Beverly, USA). Then 3 µL USER Enzyme (NEB, USA) was used with size-selected, adaptor-ligated cDNA at 37 °C for 15 minutes followed by 5 minutes at 95 °C before PCR. Then PCR was performed with Phusion High -Fidelity DNA polymerase, Universal PCR primers and Index (X) Primer. At last, the PCR products were purified (AMPure XP system) and the library quality was evaluated on Agilent Bioanalyzer 2100 system. The differently expressed genes of all comparison groups were used as the differently expressed gene set. Cluster analysis was performed on the differential genes. The RNA of 51 cases of thyroid papillary carcinoma and 4 healthy controls was analyzed by microarray (GEO datasets ID GSE27155). The gene expression data and clinical features of 458 cases of papillary thyroid carcinoma (TCGA-THCA) were downloaded from the TCGA official website (https://cancergenome.nih.gov/) for the following analysis 18.

### Immune gene interaction network and functional analysis

A protein-protein interaction (PPI) network containing TROP2 and immune genes was constructed. The immune genes were from ImmPort Shared Data (https://www.immport.org/shared/home) (PMID: 29485622), and the PPI data were from Pathway Commons (http://www.pathwaycommons.org/) (PMID: 31647099). Each protein of the screened mutation gene was a center in the PPI network. Subsequently, these proteins were filtered, and those related to immune response were classified. Kyoto Encyclopedia of Genes and Genomes (KEGG, version 90.0; www.kegg.jp) is a common online resource for interpreting biological systems from molecular level data (PMID: 27899662). A hypergeometric distribution model was used to test whether the gene modules in the network were significantly enriched in the biological pathways of KEGG database (P < 0.05).

### Immune infiltration analysis

Immune cellular fraction estimates (CIBERSORT, https://cibersort.stan ford.edu/index.php) is a deconvolution algorithm for obtaining normalized gene expression profiles (PMID: 29344893). According to the median value of TROP2 expression in TCGA database for thyroid cancer, the Wilcoxon rank sum test was used to evaluate the difference in immune cell scores between high expression group and low expression group. The significance threshold was set at P < 0.05.

### Statistical analysis

The data were analyzed using SPSS 16.0, GraphPad Prism 6 and R language (v3.2.5). The results were statistically analyzed by chi-squared test and *t*-test. P<0.05 was considered statistically significant.

## Results

### TROP2 is highly expressed in thyroid papillary carcinoma

**Figure 1:**
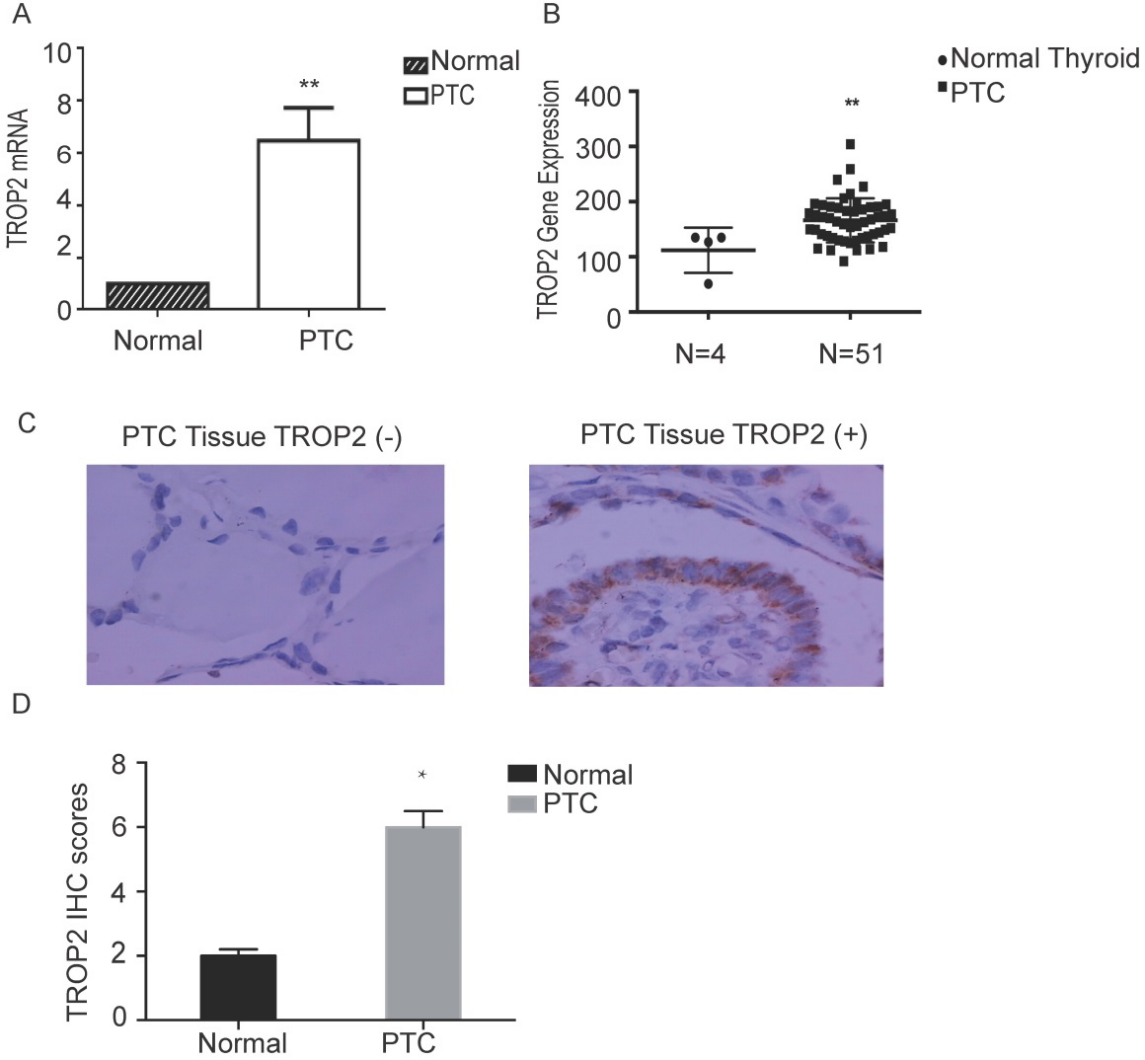
** TROP2 is highly expressed in PTC. (A)** TROP2 mRNA expression in PTC and control. **(B)** TROP2 mRNA expression in PTC and healthy controls from GEO Datasets. **(C)** TROP2 expression in PTC tissues (×400) (*P < 0.05; **P < 0.01, ***P ≤ 0.001).

The mRNA expression level of TROP2 in PTC was significantly higher than that in adjacent nontumorous thyroid tissues (P=0.04; Figure 1A). The RNA sequence results of 51 patients with thyroid papillary carcinoma and 4 healthy controls (GSE27155) were analyzed using GEO datasets. TROP2 gene expression analysis showed that TROP2 was significantly increased in PTC in comparison with those in healthy controls (Figure 1B). IHC were conducted in 60 specimens of PTC and corresponding adjacent tissues. There were 22 male and 38 female. The average age was 45.7 years (range 24-69 years). The patients were divided into 2 subgroups according to the TROP2 expression level (TROP2 positive group and TROP2 negative group). The IHC result showed that the positive expression rate of TROP2 was 68.33% in PTC tissues, while that in adjacent tissues was only 13.33% (P=0.03; Table 1 and Figure 1C). IHC results indicated that the TROP2 expression of protein in PTC is significantly higher than that in the control group (Figure 1D). Obviously, the expression of TROP2 in PTC was related to lymph node metastasis, tumor size and capsular infiltration (P<0.05), but not to sex, age, and Hashimoto's thyroiditis complications (P>0.05; Table 2).

### Depletion of TROP2 inhibits the proliferation and migration of PTC cells

**Figure 2:**
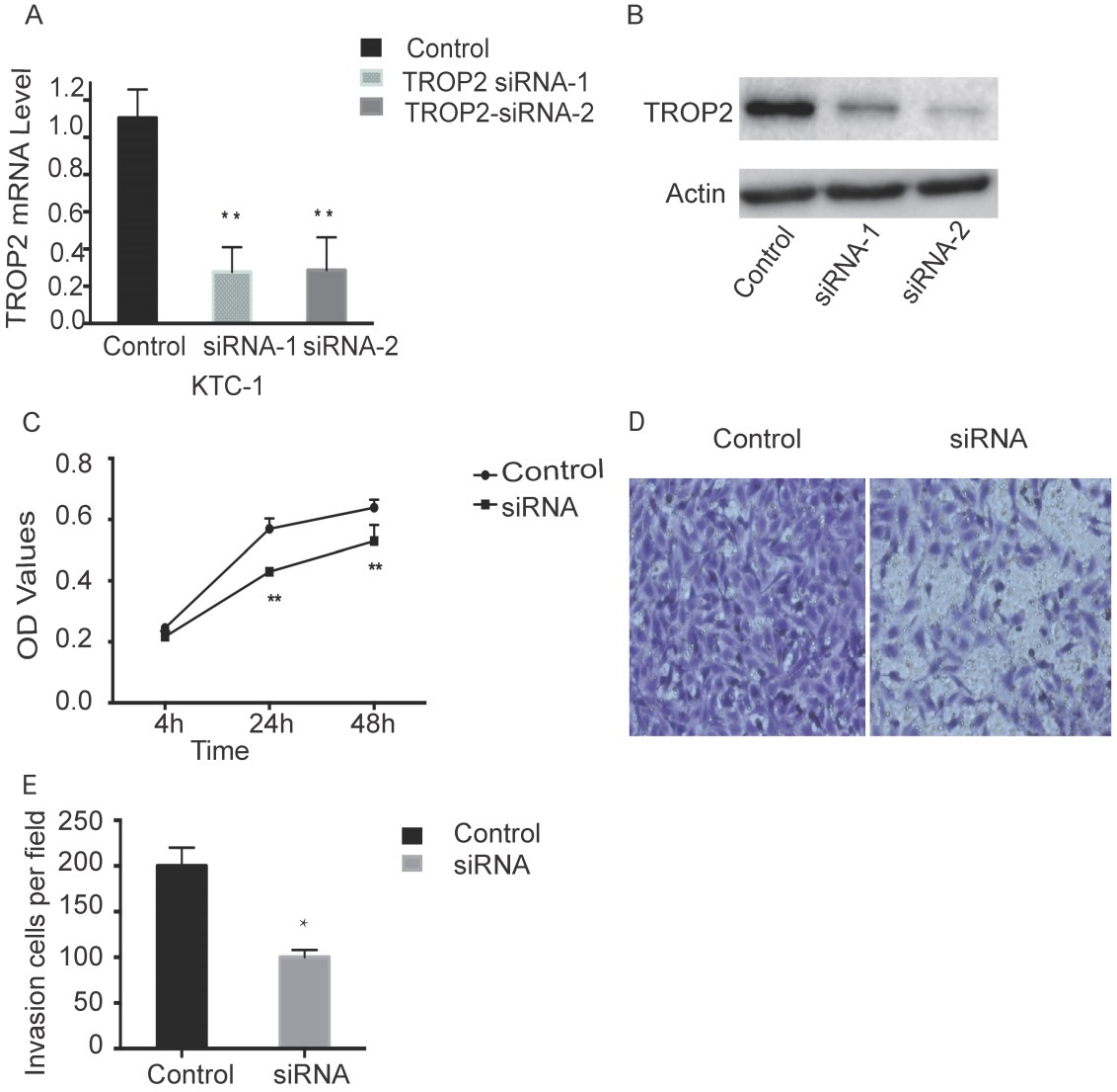
** Silencing TROP2 can inhibit proliferation of KTC-1 cells. (A)** Comparison of interference effects of TROP2 detected by PCR in KTC-1 cells. **(B)** Comparison of interference effects of TROP2 detected by Western-blotting in KTC-1 cells. **(C)** The effect of TROP2 silencing on proliferation of KTC-1 cells was determined by transwell assay. **(D and E)** The cell numbers of TROP2 control and siRNA KTC-1 cells by transwell assay. Statistically significant differences are marked by an asterisk (*P < 0.05; **P < 0.01, ***P ≤ 0.001).

TROP2 siRNA could significantly down-regulate the expression of TROP2 in KTC-1 cells (P<0.05; Figure 2A-B). The MTT assay results indicated proliferation of TROP2 siRNA KTC-1 cells was significantly lower than that of the control group (P<0.05; Figure 2C). Wounding healing assay and Transwell assay confirmed that after inhibiting the expression of TROP2, the migration of KTC-1 cells was significantly lower than that of the control group (P<0.05; Figure 2D-E, 3A-B). The transfection efficiency exceeded 90% in CGTHW-3 cells (Figure S1A). TROP2 siRNA could down-regulate the expression of TROP2 with the mRNA and WB levels (P<0.05; Figure S1B-C). The MTT assay results indicated the proliferation of TROP2 siRNA CGTHW-3 cells was significantly lower than that of the control group (P<0.05; Figure S1D). The results of the plate clone experiment were also similar to the MTT assay results (P<0.05; Figure S1E-F). Wounding healing assay and Transwell assay indicated that the aggressiveness of TROP2 siRNA CGTHW-3 cells was significantly lower than that of the control groups (P<0.05; Figure S2A-C).

### TROP2 and ISG15 correlation analysis in PTC

**Figure 4:**
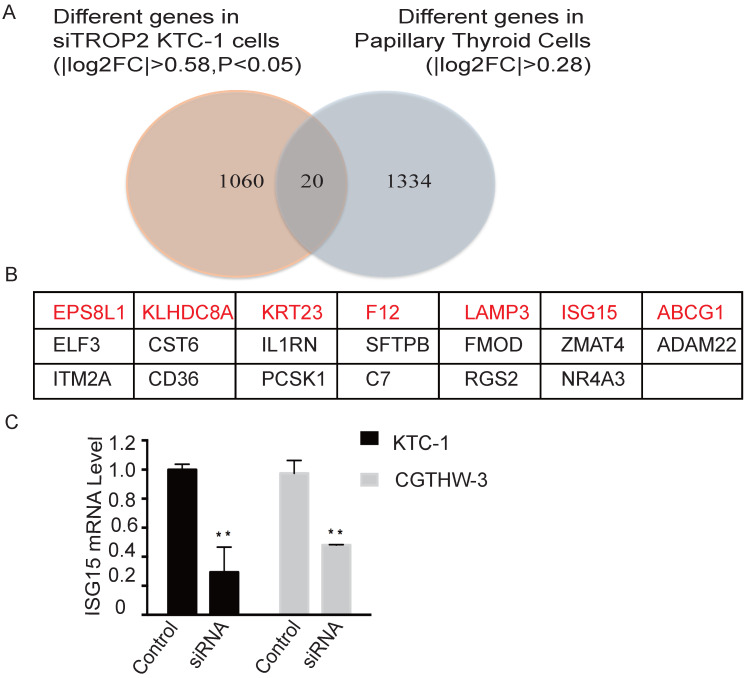
** TROP2 and ISG15 expression were significantly correlated in PTC patients. (A)** Overlap of different genes in TROP2 siRNA KTC-1 cells and PTC. **(B)** The gene list of 20 overlapped common genes. The genes in red indicated that the genes were up-regulated in PTC and down-regulated in TROP2 siRNA KTC-1 cells. **(C)** ISG15 was down-regulated in TROP2 siRNA cells. Statistically significant differences are marked by an asterisk (*P < 0.05; **P < 0.01, ***P ≤ 0.001).

RNA sequencing was performed on TROP2 and negative siRNA KTC-1 cells. The differently expressed genes in each control group were considered as the differently expressed gene sets. Cluster analysis was also conducted (Figure S3). There were 20 genes differently expressed in TROP2 siRNA KTC-1 in comparison with negative siRNA KTC-1. In addition, 20 genes were expressed differently in PTC in comparison with healthy controls (GSE27155; Figure 4A). Among them, Epidermal growth factor receptor pathway substrate 8-related protein1 (EPS8L1), Kelch domain containing protein 8 (AKLHDC8A), Recombinant Keratin 23 (KRT23), Coagulation factor XII (F12), Lysosome-associated membrane glycoprotein 3 (LAMP3), Interferon-stimulated gene 15 (ISG15), ATP-binding cassette superfamily G member 1 (ABCG1) were up-regulated in PTC and down-regulated in TROP2 siRNA KTC-1 cells (Figure 4B). The above seven genes were further verified by PCR in KTC-1 and CGTHW-3 cell lines. The results indicated that EPS8L1, KLHDC8A, KRT23, F12, LAMP3, ABCG1 were lowly expressed in PTC cell lines, which was difficult to detect. Only ISG15 was significantly decreased in TROP2 siRNA KTC-1 and CGTHW-3 cells (Figure 4C).

To further investigate the relationship between TROP2 and ISG15 expression in PTC, the RNA sequence results of 51 patients with PTC and 4 healthy controls in the public database (GSE27155) were analyzed. The TROP2 and ISG15 genes were expressed as follows (Figure S4A-B). The results indicated that the expression mRNA level of ISG15 was higher in PTC samples with high expression of TROP2 and lower in PTC samples with low expression of TROP2 (P<0.001; Table 3). Spearman rho value was 0.496 (P<0.001), suggesting a moderate correlation between the expression of TROP2 and ISG15 in PTC patients. To verify the finding in different databases, the expression of TROP2 and ISG15 in 458 PTC patients in TCGA database were further analyzed. The results showed that PTC patients with high expression of ISG15 and TROP2 accounted for 88.8% of the total patients. Additionally, 96.5% of PTC patients with low expression of ISG15 had low TROP2 expression (P<0.001; Table 4). Spearman Rho value was 0.803 (P<0.001), suggesting a strong positive correlation between the expression of TROP2 and ISG15 in PTC patients. The results of TROP2 and ISG15 expression in PTC patients from TCGA database were consistent with those from GEO DataSets.

### Immune interaction and Enrichment pathway analysis of TROP2 expression

**Figure 5:**
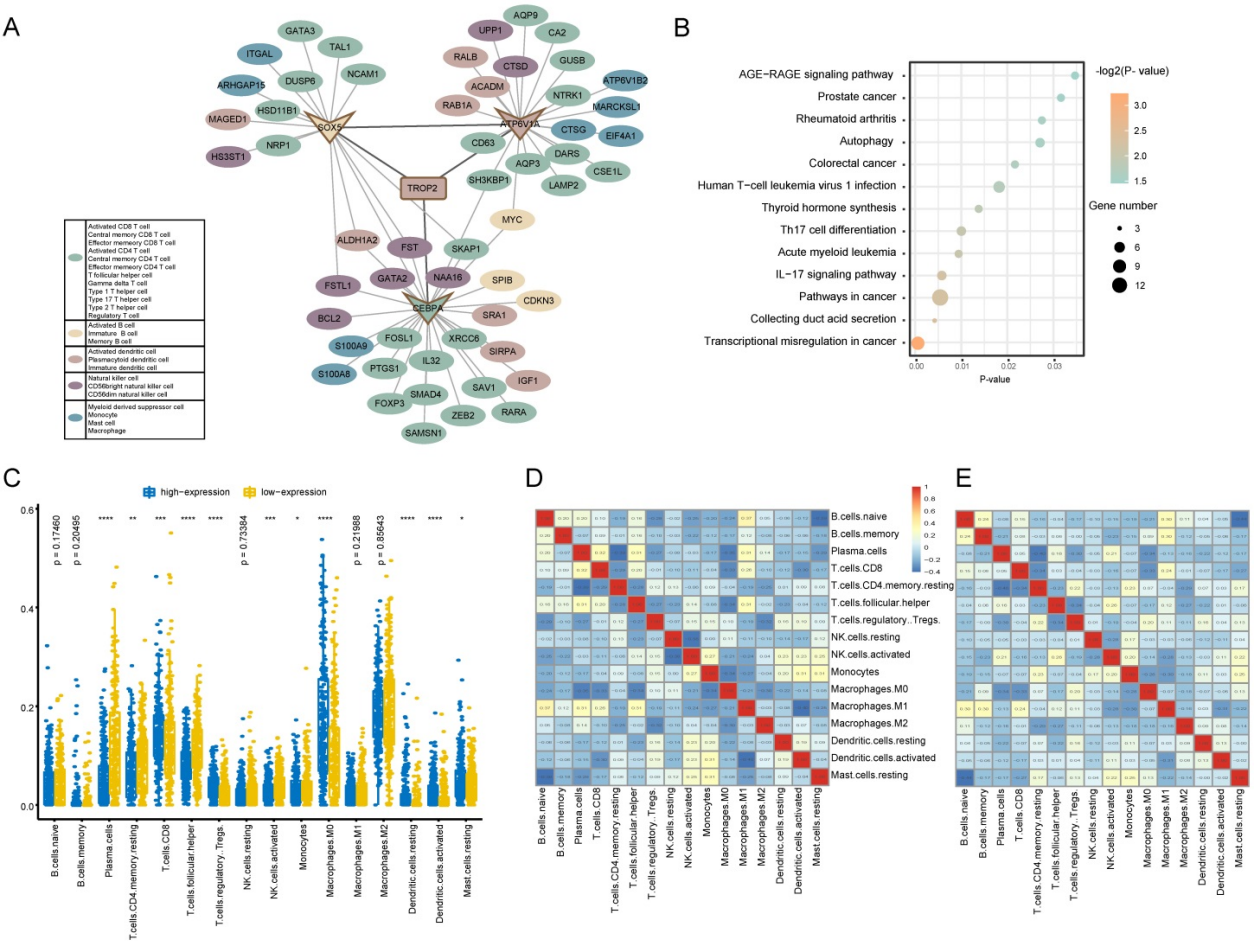
** Immune function analysis of TROP2 expression. (A)** TROP2-immune PPI network with immune-related genes. Each color represents a subset of immune cells. **(B)** KEGG pathway analysis of genes associated with immune. **(C)** Immune infiltration difference between TROP2 high and low expression. **(D, E)** Correlation matrix of immune cell proportions. D: The correlation matrix of immune infiltration of TROP2 high expression samples, E: the correlation matrix of immune infiltration of TROP2 low expression samples.

In order to reveal the regulatory mechanism of TROP2 in the tumor immune microenvironment, a TROP2 immune PPI network was constructed (Figure 5A). The results showed that the PPI network was composed of 69 Hippo-immune relationships, including 57 immune genes. Among them, TROP2 interacts directly with ATP6V1A, CEBPA and SOX5. The network further explored the immune genes directly interacting with ATP6V1A, CEBPA and SOX5. It was found that among the immune genes adjacent to one-step or two-step of TROP2, the number of genes belonging to T cell type was the largest. KEGG enrichment analysis was used to further explore the biological functions of immune genes related to TROP2 (Figure 5B). Transcriptional misregulation and thyroid hormone synthesis pathways related with thyroid cancer, as well as immune-related pathways such as IL-17 signaling pathway and Th17 cell differentiation were significantly enriched.

### Tumor-infiltrating immune cells associated with *TROP2* expression

The association between TROP2 expression and tumor-infiltrating immune cells in thyroid cancer microenvironment was evaluated using CIBERSORT algorithm. More than 80% of the immune cell types with an immune score of 0 were removed.

As shown in Figure 5C, the results showed that the immune infiltration score of 16 immune cell types were different between high expression and low expression groups. Moreover, it was observed that activated memory CD8 T cells and macrophager M0 cells were more enriched in the high expression group, while plasma cells and follicular helper T cells were more enriched in the low expression group. Furthermore, the result from the correlation matrix revealed that there was a correlation between immune cells with high or low expression of TROP2 (Figure 5D-E).

## Discussion

The incidence rate of PTC is about 0.5-10 per 100,000 populations worldwide 19. It is well known that most PTC patients have relatively good prognosis. However, the incidence rate of this disease has been increasing in recent years, and has a certain impact on the health of the residents. Age directly affects the stage and prognosis of PTC. Currently, there is no other effective treatment except surgery and radiation therapy. The prognosis of PTC is poor if envelope or lymph node metastasis occurs 20. With the continuous progress of medical technology, gene therapy has brought good news to many tumor patients. The further determination of PTC specific molecular markers may provide new directions and targets for the treatment of PTC.

Many studies have confirmed that the TROP2 protein is highly expressed in a variety of head and neck tumors. In our study, it was found that the expression level of TROP2 in PTC was significantly higher than that of adjacent normal thyroid tissues, suggesting that the expression of TROP2 gene is related to PTC. The immunohistochemistry results of this experiment further showed that the positive expression rate of TROP2 protein in PTC was 68.33%, while the positive expression rate of TROP2 protein in adjacent tissues was 33.33% (P<0.05). In our study, PCR experiments further confirmed that the relative expression level of TROP2 protein in PTC was significantly higher than that in the control group, which was consistent with the immunohistochemical results. It was confirmed again that the expression level of TROP2 protein in PTC was significantly higher, and the difference was statistically significant (P<0.05). Immunohistochemistry analysis indicated that the high expression of TROP2 protein was associated with lymphatic metastasis, tumor size and envelope infiltration of PTC. It is suggested that the high expression of TROP2 protein may be related to the prognosis of PTC.

Studies have confirmed that high expression of TROP2 is not only found in solid tumors such as liver cancer, breast cancer, lung cancer, gastric cancer, oral cancer, thyroid carcinoma, but also affects the development and prognostic of these patients 21-28. And it plays an important role in proliferation, invasion and metastasis of tumor cells. This study showed that the expression of TROP2 was significantly up-regulated in PTC. Further, TROP2 expressions were down-regulated by small interference RNA. The proliferation of TROP2 siRNA CGTHW-3 and KTC-1 thyroid cancer cells decreased significantly, and the migration ability of TROP2 siRNA PTC was also significantly weakened. The signal pathway mediated by TROP2 in thyroid cancer needs to be further studied. TROP2 protein expression mediated integrin β-1-RACK1-Src and FAK signaling pathways regulate cell adhesion, lymph node metastasis and diffusion. However, the specific biological mechanisms are still unclear and needs to be further discussed.

ISG15 (Interferon-stimulated gene 15) is an ubiquitinated protein 29. Its structure is similar to that of ubiquitin and can be covalently modified by connecting with other proteins. ISG15 can be induced by interferon and cross-react with ubiquitin protein. The expression of ISG15 can be promoted by external stimuli, such as bacterial and viral infection and tumorigenesis.

USP18 enhances the anticancer effect of tumor suppressor protein PTEN by affecting the covalent modification of ISG15 30. In prostate cancer cells, covalent modification of ISG15 is weakened or inhibited to inhibit the proliferation of cancer cells 31. In breast cancer, the expression of estrogen receptor ER, progesterone receptor PR and human epidermal growth factor receptor HER-2 promotes the occurrence and development of breast cancer through DTX3L-ISG15 signaling pathway 32, 33. In hepatoma cells, the knockout of E3ligase HERC5 can reduce the covalent modification of ISG15 by p53, so as to reduce the degradation of P53 by 20S protease and inhibit the occurrence of tumor 34.

The results showed that ISG15 decreased significantly in TROP2 KTC-1 cells. The expression level of ISG15 in PTC patients was also significantly higher than that in healthy controls. Correlation analysis showed that there was a significant correlation between the expression of TROP2 and ISG15 in PTC patients. It is suggested that TROP2 may promote the growth and metastasis of PTC by regulating ISG15, which providing a new idea for the treatment of patients with PTC. Yuan HG *et al.* proposed that ISG15 can promote the proliferation, invasion and migration of esophageal squamous cell carcinoma via c-MET/Fyn/β-catenin pathway 35. *Li XX et al.* reported that TROP2 may promote the proliferation, migration and metastasis of gallbladder cancer cells by regulating PI3K/AKT pathway and inducing EMT 36. However, the specific mechanism of the interaction between TROP2 and ISG15 and the specific role of ISG15 in the occurrence and development of PTC still need to be further explored.

In this study, the TROP2-immune PPI network results showed that TROP2 interacted with ATP6V1A, CEBPA and SOX5. These immune genes belong to the T cells. In addition, the PTC is associated with the activation of immune pathways, such as IL-17 signaling pathway and Th17 cell differentiation. Obviously, the immune system of PTC patients was activated. Zhen* YX et al.* 37 showed the number of immune cells promoting PTC formation increased significantly, such as M2 macrophages, M0 macrophages, Tregs and neutrophils. They further suggested that immune cell infiltration is associated with more BRAF mutations and fewer RAS mutations. It was also found that TROP2 was related to activated memory CD8 T cells and macrophager M0 cells plasma cells and T cell follicular helper cells in this work. PTC may be related to chronic inflammation. Immune escape is an important mechanism of PTC. Immune cells play different roles in tumor microenvironment. Single cell analysis can be used to discover the role of various immune cells in tumor formation 38. The increase of Tregs cells promotes the formation and metastasis of tumor and is associated with the prognosis. Lin P *et al.* 39 showed that the immune cell infiltration was correlated with age, tumor size, tumor stage, metastasis and number of tumor. The relationship between immune cells and tumor cells will be further revealed. Lu HS *et al.* 40 showed that CD8+CD38+ and TNF-α in PTC increased significantly. CD8+CD38+ is related to lymph node metastasis. They may be useful biomarkers of PTC. In recent years, many genes regulating immune cell function have been founded. Immunotherapy has attracted more and more attention. New immunodetection points need to be further explored in the future. However, the mechanism of immune cells in PTC is not clear. It also provides a new opportunity to explore the pathogenesis of PTC and find new therapeutic targets.

In summary, TROP2 was found to be highly expressed in PTC and promoted the proliferation, invasion and migration of PTC. Moreover, it participated in the regulation of immune cells and promoted tumor progression. Additionally, ISG15 decreased significantly in TROP2 siRNA PTC cells and increased significantly in PTC patients. Therefore, TROP2 and ISG15 expression were significantly correlated in PTC patients. Our study provides new insights into the new molecular mechanisms of TROP2 in PTC.

## Supplementary Material

Supplementary figures.

## Figures and Tables

**Figure 3:**
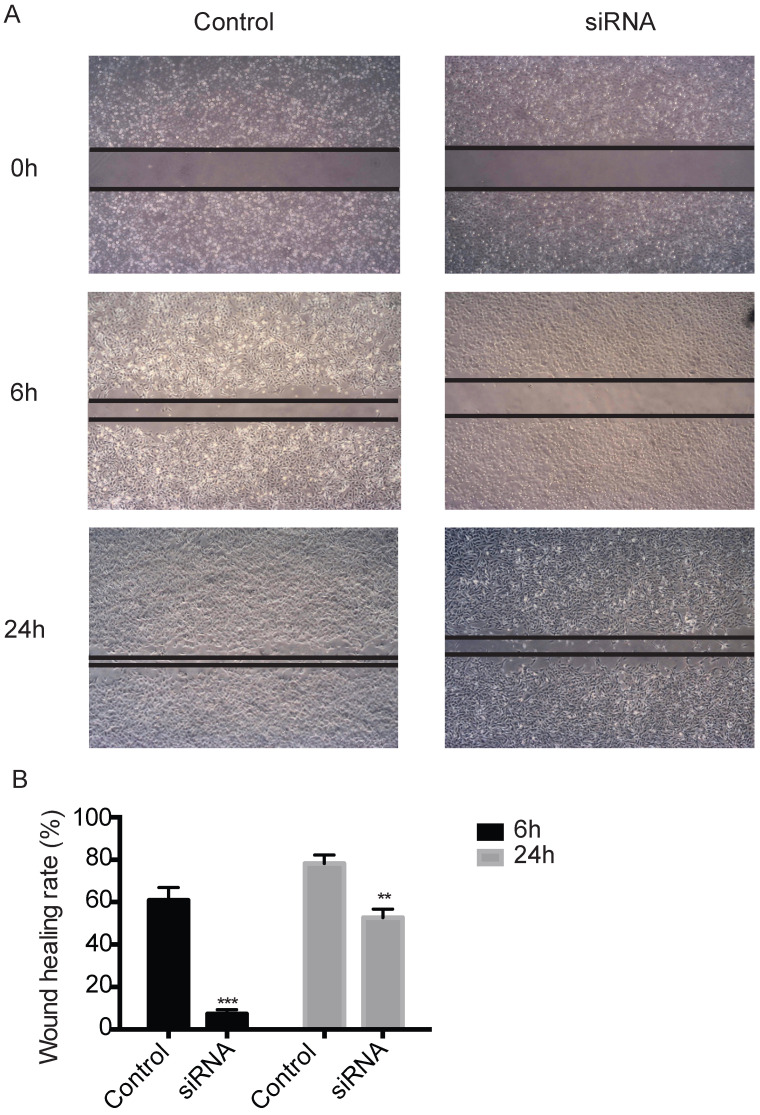
** Silencing TROP2 can inhibit migration of KTC-1 cells. (A)** Effects of TROP2 silencing on migration of KTC-1 cells detected by wound healing assays. **(B)** Wound healing rates of TROP2 control and siRNA KTC-1 cells. Statistically significant differences are marked by an asterisk (*P < 0.05; **P < 0.01, ***P ≤ 0.001).

**Table 1:** Comparison of TROP2 expression between PTC and control

Group	Positive	Negative	χ²	P
PTC	41	19	37.56	0.03
Control	8	52		

**Table 2:** Clinical and molecular characteristics of PTC patients enrolled in immunohistochemical research

Clinical characteristics	N	TROP2 positive (%)	χ²	P value
**Sex**				
Male	22	15 (68.18)		
Female	38	26 (68.42)	0.072	0.078
**Age (year)**				
≥45	36	25 (69.44)		
<45	24	16 (66.67)	0.776	0.083
**Tumor size (cm)**				
≥2	24	21 (87.5)		
<2	36	20 (55.56)	5.395	0.021
**Tumor Stage**				
I-II	24	19 (79.17)		
III-IV	36	22 (61.11)	1.415	0.234
**Lymph Metastasis**				
Yes	24	23 (95.83)		
No	36	18 (50.00)	11.942	0.001
**Capsular Invasion**				
Yes	32	27 (84.38)		
No	28	14 (50.00)	6.644	0.010
**Hashimoto's Thyroiditis**				
Yes	24	16 (66.67)		
No	36	25 (69.44)	0.003	0.955

**Table 3:** Correlations of TROP2 and ISG15 expression in PTC from GEO Datasets

TROP2 expression	ISG15 expression		Total	P value
	High expression	Low expression		
High expression	27	7	34	<0.001
Low expression	5	12	17	
Total	32	19	51	

High TROP2 expression group: TROP2 expression value (quantile-normalized and then log-transformed with base 10 logarithms) ≥4; low TROP2 expression group: TROP2 expression value (quantile-normalized and then log-transformed)<4; high ISG15 expression group: ISG15 expression value (quantile-normalized and then log-transformed with base 10 logarithms)≥3.1; low ISG15 expression group: ISG15 expression value (quantile-normalized and then log-transformed)< 3.1.

**Table 4:** Correlations of TROP2 and ISG15 expression in PTC from TCGA

TROP2 expression	ISG15 expression		Total	P value
	High expression	Low expression		
High expression	80	10	90	<0.001
Low expression	13	355	368	
Total	93	365	458	

High TROP2 expression group: TROP2 expression value ≥ 440; low TROP2 expression group: TROP2 expression value < 440; high ISG15 expression group: ISG15 expression value ≥10; low ISG15 expression group: ISG15 expression value < 10.
