# Supplementary material for: TROP2 modulates the progression in papillary thyroid carcinoma
Source: J Cancer. 2021 Sep 27;12(22):6883–93. doi: 10.7150/jca.62461 (PMC8518010; doi:10.7150/jca.62461)
Supplement: Supplementary file 1 — Supplementary figures. [file jcav12p6883s1.pdf]

Figure S1 Sun. et al.

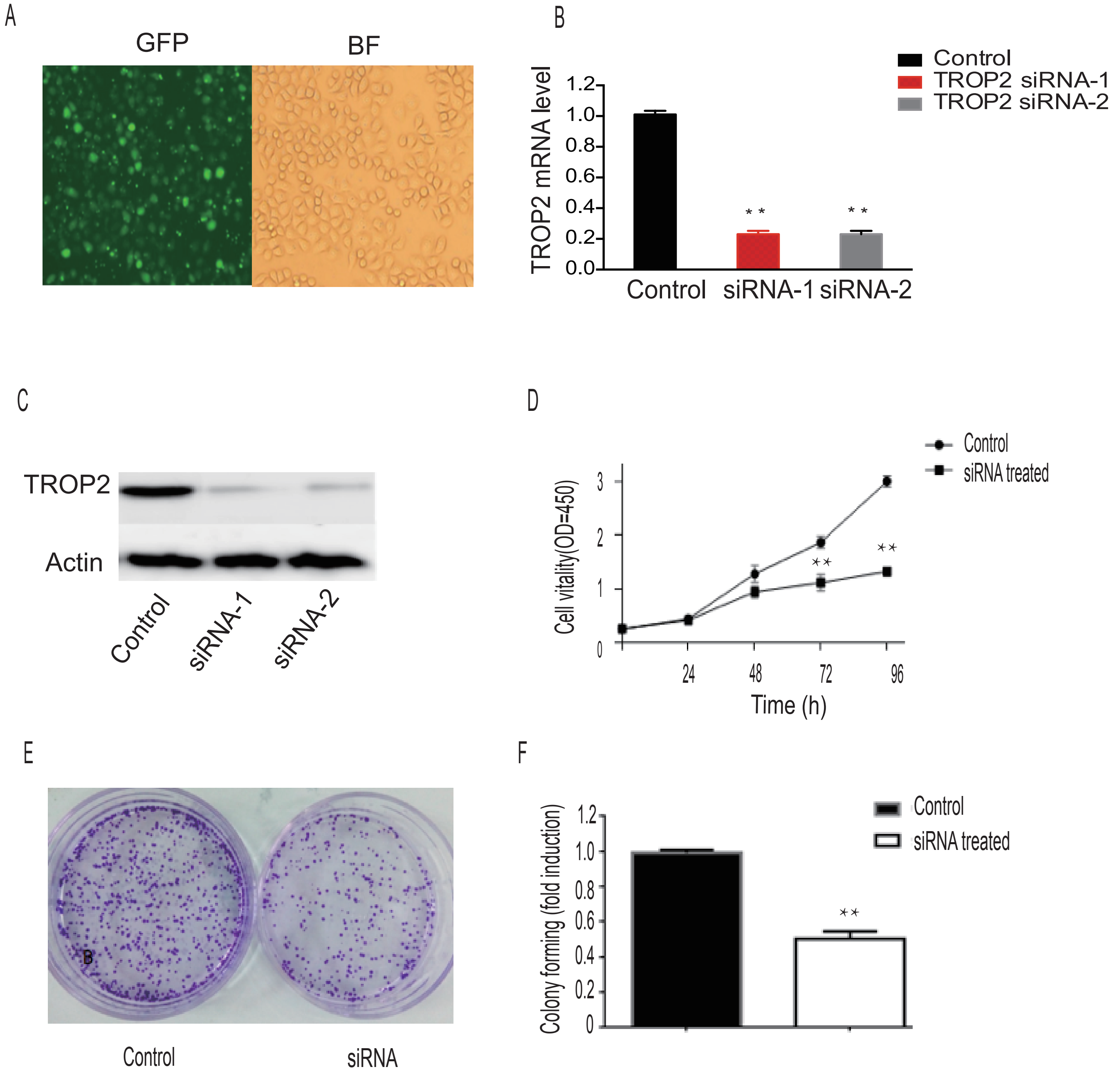

Figure S1 Silencing TROP2 can inhibit proliferation of CGTHW-3 cells. (A) Detection of liposome transfection efficiency. (B) Comparison of interference effects of TROP2 detected by PCR in CGTHW-3 cells. (C) Comparison of interference effects of TROP2 detected by Western-blotting in CGTHW-3. (D) Effects of TROP2 on proliferation of CGTHW-3 cells detected by MTT assays. (E) Effects of TROP2 on proliferation of CGTHW-3 cells by colony-forming unit assays. (F) Number of colony-forming unit of TROP2 siRNA and control CGTHW-3 cells. Statistically significant differences are marked by an asterisk (*P < 0.05; **P< 0.01, ***P≤ 0.001).

Figure S2 Sun. et al.

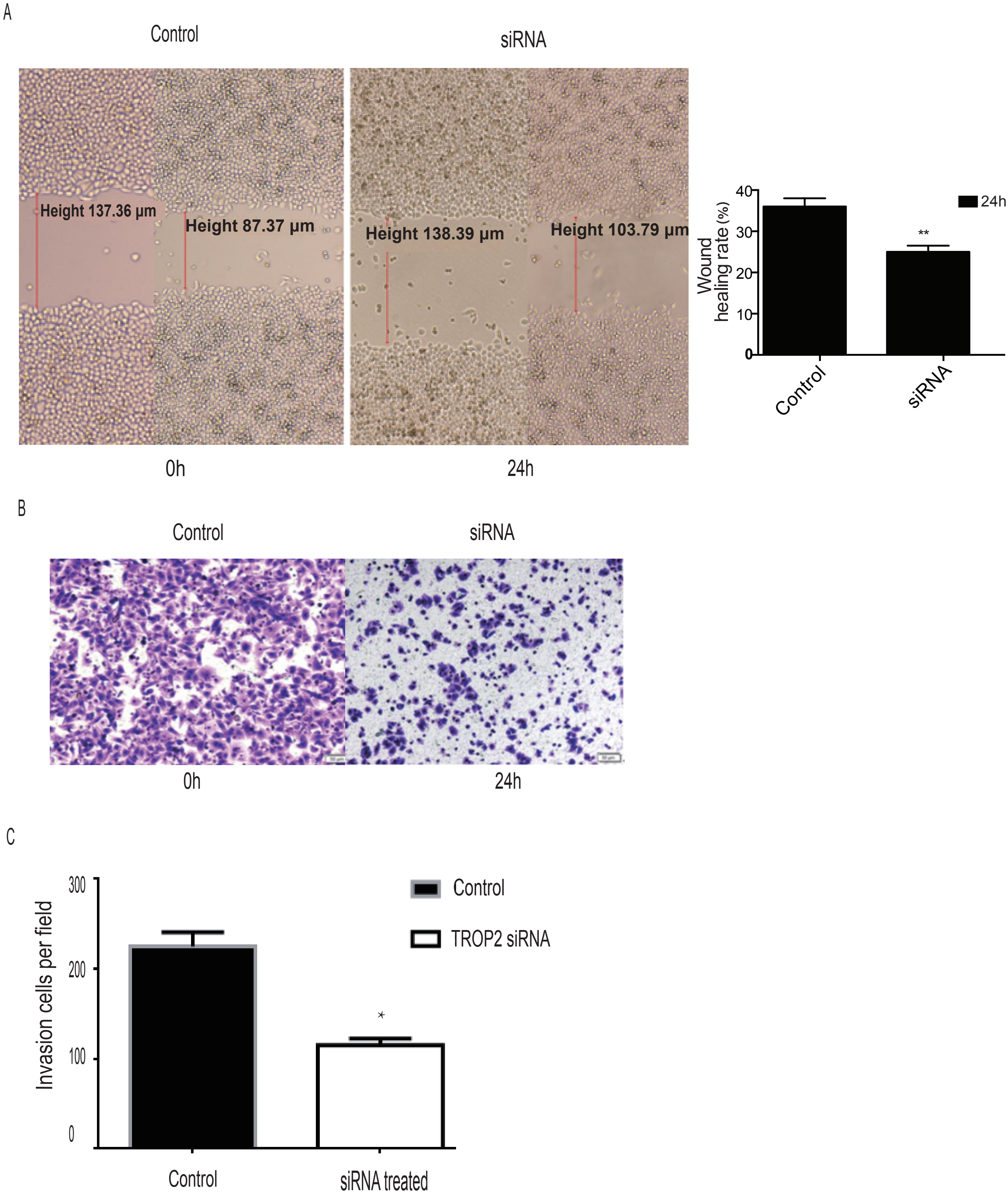

Figure S2. Silencing TROP2 can inhibit migration of CGTHW-3 cells. (A) The effect of TROP2 silencing on migration of CGTHW-3 cells by wounding healing assays. (B) The effect of TROP2 silencing on migration of CGTHW-3 Cells by transwell assays. (C) The cell numbers of TROP2 control and siRNA CGTHW-3 cells by transwell assay. Statistically significant differences are marked by an asterisk (*P < 0.05; **P< 0.01, ***P≤ 0.001).

Figure S3 Sun. et al.

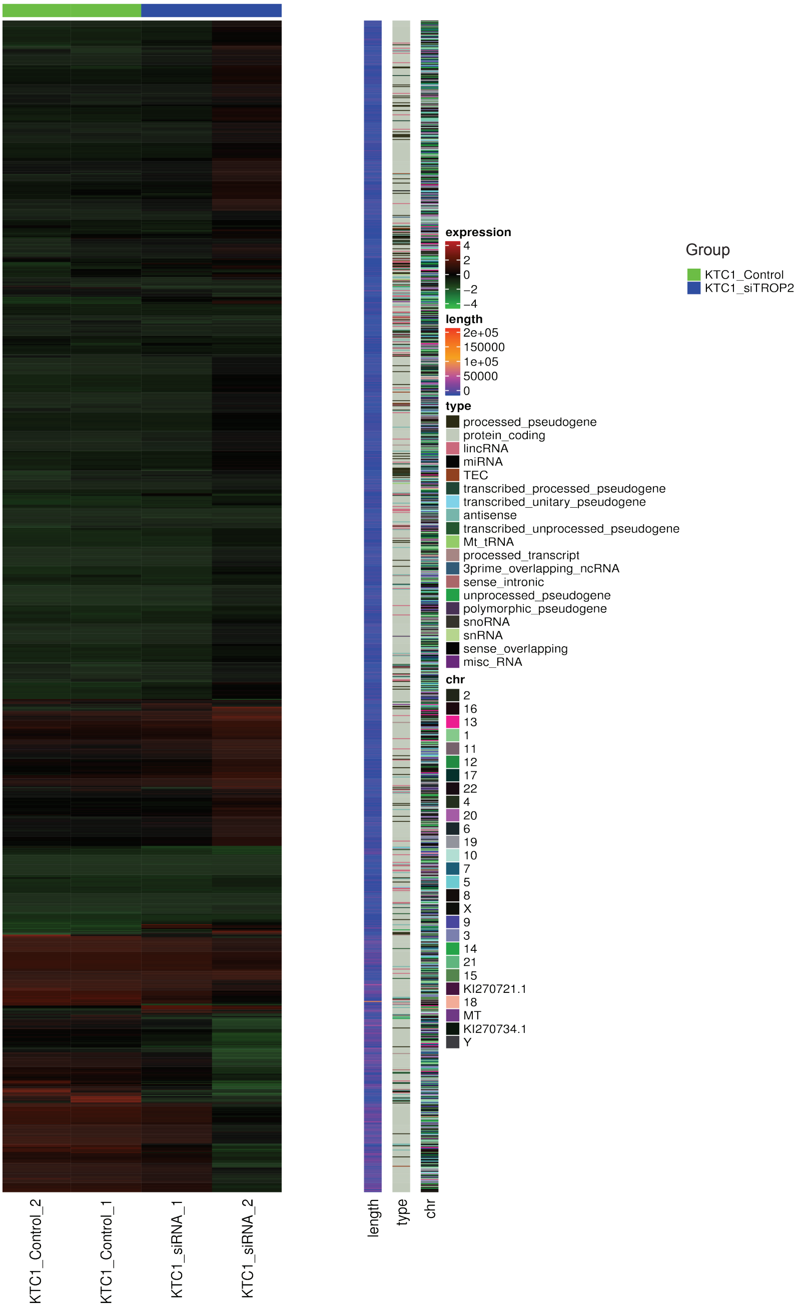

Figure S3. Gene Clustering Heat Map of TROP2 siRNA and control KTC-1 cells.

Figure S4 Sun et.al.

A TROP2

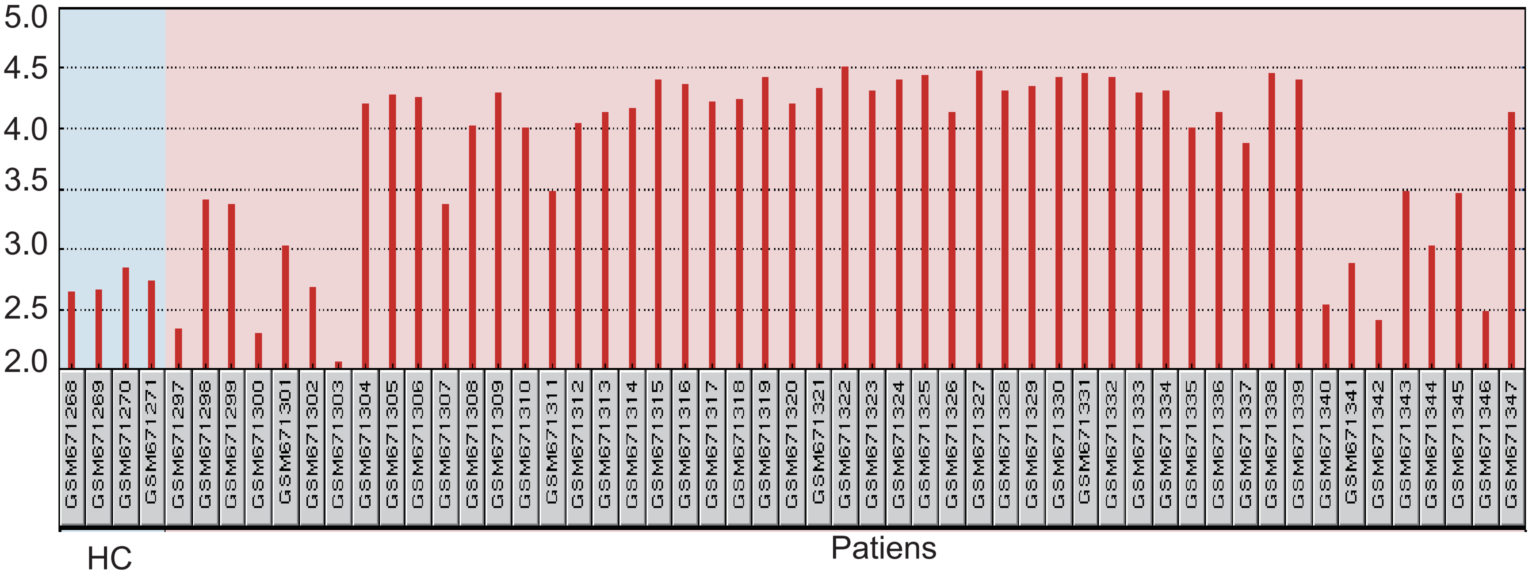

B ISG15

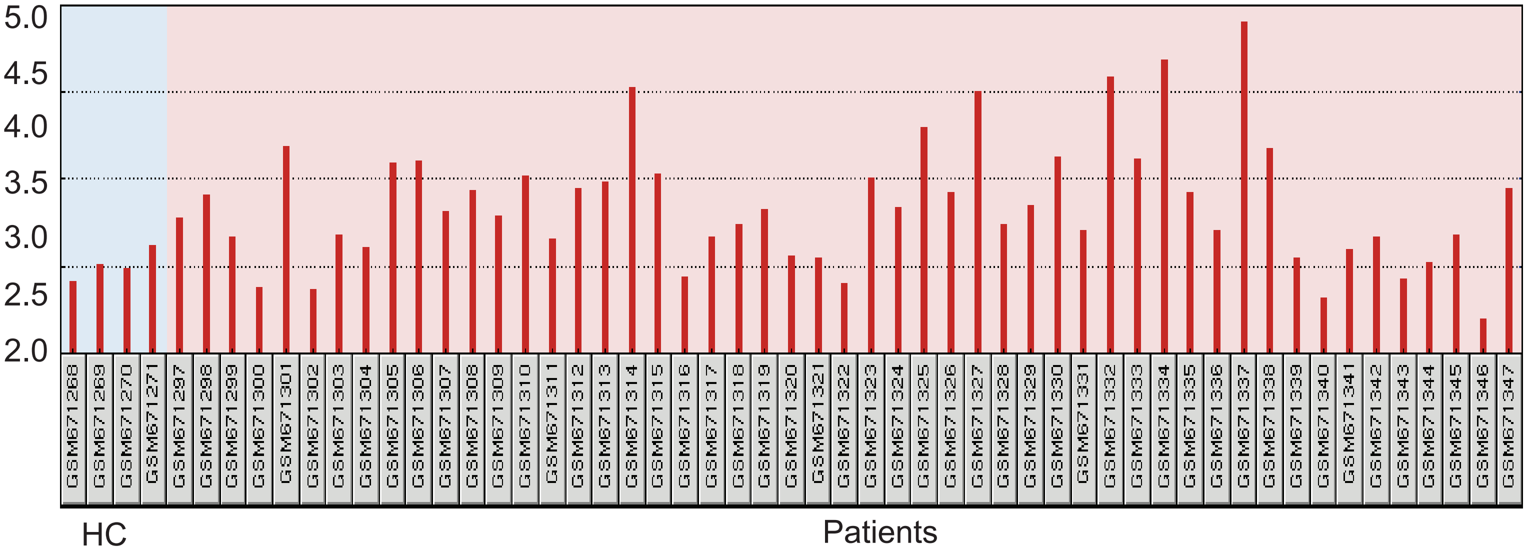

Figure S4. TROP2 and ISG15 exprssion in PTC patients and healthy control from GEO Datasets. (A) Expression of TROP2 in PTC patients and healthy controls. (B) Expression of ISG15 in PTC patients and healthy controls.
